# Voting, deliberation and truth

**DOI:** 10.1007/s11229-016-1268-9

**Published:** 2016-11-29

**Authors:** Stephan Hartmann, Soroush Rafiee Rad

**Affiliations:** 1 Munich Center for Mathematical Philosophy, LMU Munich, Geschwister-Scholl-Platz 1, 80539 Munich, Germany; 2 Institute for Logic, Language and Computation, University of Amsterdam, P.O. Box 94242, 1090 GE Amsterdam, The Netherlands

**Keywords:** Rational deliberation, Voting, Group consensus, Deliberation model

## Abstract

There are various ways to reach a group decision on a factual yes–no question. One way is to vote and decide what the majority votes for. This procedure receives some epistemological support from the Condorcet Jury Theorem. Alternatively, the group members may prefer to deliberate and will eventually reach a decision that everybody endorses—a consensus. While the latter procedure has the advantage that it makes everybody happy (as everybody endorses the consensus), it has the disadvantage that it is difficult to implement, especially for larger groups. Besides, the resulting consensus may be far away from the truth. And so we ask: Is deliberation truth-conducive in the sense that majority voting is? To address this question, we construct a highly idealized model of a particular deliberation process, inspired by the movie *Twelve Angry Men*, and show that the answer is ‘yes’. Deliberation procedures can be truth-conducive just as the voting procedure is. We then explore, again on the basis of our model and using agent-based simulations, under which conditions it is better *epistemically* to deliberate than to vote. Our analysis shows that there are contexts in which deliberation is epistemically preferable and we will provide reasons for why this is so.

## Introduction

Consider a group that has to decide on a a factual yes–no question. A jury in court, for example, has to decide whether the defendant is guilty or not. An environmental committee has to decide on a certain policy recommendation for the government. Situations like these raise the following questions: (1) Which decision-making procedure should be applied here? (2) What justifies the chosen decision-making procedure in general? And: (3) What justifies the application of the chosen decision-making procedure in the specific situation?

There are three types of criteria to evaluate a proposed decision-making procedure: First, there are *practical reasons*: The procedure should be easy to implement; it should be implementable for groups of a given size; and following the procedure should not take too much time to arrive at a decision. Second, there are *procedural reasons*: The procedure should implement certain principles of rational decision-making; it should be fair; and it should end up in a consensus that all group members endorse. Third, there are *epistemic reasons*: The group decision should be reliable and it should coincide with the fact of the matter if it is the task of the group to decide on a fact of the matter (e.g. the group decision should be ‘guilty if and only if the defendant is guilty). Unfortunately, there is no decision-making procedure that scores highest on each of these criteria. Hence, in any given decision-making situation some compromise has to be made.

Let us now introduce two decision making procedures and their advantages and disadvantages. First, there is **majority voting**. Here each group member casts a vote, and the group decision is the one that the majority (or a supermajority) of group members supports. This procedure ranks high on practical grounds: It is easy to implement, it works for groups of large size, and it does not take long to arrive at a group decision. There are also strong epistemic reasons in favor of majority voting: Most relevant here is the Condorcet Jury Theorem which considers a group of *n* independent voters each of whom has a probability greater than 1/2 to make the right decision. It then follows (from the Weak Law of Large Numbers) that the probability that the majority makes the right decision converges to one if *n* goes to infinity.^1^ Hence, majority voting is a reliable procedure if one is interested in tracking the truth. Concerning procedural reasons, majority voting does not fare too well: The procedure can leave almost half of the group unhappy, which may be considered as unfair. it also does not result in a consensus that everyone endorses.

Second, there are **deliberation procedures**. These procedures are more dynamical than the voting procedure. Here the group members argue for their verdict, they try to convince each other, they may learn from each other and change their mind as a result of this. If all goes well, a deliberation procedure results in a consensus, i.e. in a decision that everyone supports and endorses. It lies in the nature of deliberation procedure that they do not follow a strict rule. For example, there is usually no given order in which the various group members speak, and there are no rules that govern the belief change dynamics. There is not just one deliberation procedure, there are many and every deliberation is in a way special. This makes it harder to evaluate deliberation procedures according to the criteria listed above. But let us try. Deliberation procedures are certainly not optimal on practical grounds. It is impossible to implement them for larger groups (think about the citizens of a country) and it may take very long until the group reaches a consensus (if it reaches a consensus at all). The main advantage of deliberation procedures is procedural: It is good that all group members have a chance to actively participate in the decision making process, that the group productively interacts, and that everyone endorse the resulting consensus. Finally, there is a large literature which defends the view that deliberation is also epistemically advantageous. Here it is stressed that the collective evaluation of arguments increases the chance of identifying errors and that the chances of manipulation are lower in this case as the group controls the flow of information.^2^

There is, however, no formal analysis which (i) shows that deliberation procedures are truth-conducive (in a similar way as majority voting is truth conducive according to the Condorcet Jury Theorem) and that (ii) explores which procedure does better (under certain conditions) epistemically. We will address these question in this article by constructing and analyzing a simple and highly idealized model of deliberation. It remains an open question whether the results we obtain also hold for more detailed and realistic models. The proposed model will be a good starting point for further studies.

The remainder of this article is organized as follows. Section 2 presents and motivates our model of deliberation. Section 3 explores the consequences of the model and shows that deliberation is, under certain conditions, truth conducive. Section 4 compares the probability of making the right decision using majority voting with the probability of making the right decision using deliberation. Finally, Sect. 5 concludes and suggests a number of questions which should be addressed in future research.

## A Bayesian model of deliberation

In this section, we first formalize the voting procedure and its probabilistic analysis (Sect. 2.1). Then we introduce our new model of deliberation (Sect. 2.2). As we want to compare the two procedures, we make sure that the parameters that characterize the epistemic performance of the agents in the voting procedure also show up in the model of deliberation. Our model of deliberation is Bayesian, i.e. we assume that the group members have partial beliefs about the truth or falsity of some hypothesis (e.g. ‘the defendant is guilty’) on the basis of which they cast a (first) vote. In the course of deliberation, they then update their beliefs, taking the (previous) votes of the other group members into account and using Bayes Theorem. Our models specifies the details of this procedure.

### The voting procedure

We consider a group of *n* members, denoted by $a_{1},\ldots ,a_{n}$, who deliberate on the truth or falsity of some hypothesis. Throughout this article, we assume that *n* is an odd number. To proceed we introduce a binary propositional variable *H* with the values, H: the hypothesis is true, and $\lnot$H: the hypothesis is false. For reasons of symmetry that will become apparent immediately, we assume that the hypothesis is true. The group members express their individual verdicts in terms of a yes/no vote. The votes are represented by binary propositional variables $V_i$ (for $i=1,\dots ,n$) with the values: V$_i$: Group member $a_i$ votes that the hypothesis is true, and $\lnot$V$_i$: Group member $a_i$ votes that the hypothesis is false.

Next, we make two assumptions: First, we assume that the votes are independent, given the truth or falsity of the hypothesis, i.e.1Second, we assume that each group member $a_i$ is partially reliable with a *first order reliability* $r_i$ defined as follows:

2 $$\begin{aligned} r_i := P(\mathrm{V_i \vert H}) = P(\mathrm{\lnot V_i \vert \lnot H}). \end{aligned}$$

Here we assume that the rate of false positives equals the rate of false negatives. This assumption can, of course, be easily relaxed.

We can now calculate the probability that the majority makes the right judgment:

3 $$\begin{aligned} P_V = \sum _{k=\frac{n+1}{2}}^{n} \sum _{\mathop {\subset \{a_{1},\ldots ,a_{n}\}}\limits ^{\{a_{j_{1}},\ldots ,a_{j_{k}}\}} }\prod _{\mathop {\{j_{1},\ldots ,j_{k}\}}\limits ^{t \in }}r_{t}\prod _{\mathop {\{j_{1},\ldots ,j_{k}\}}\limits ^{t \notin }}(1-r_{t}) \ . \end{aligned}$$

If all group members are equally likely to make the right individual judgment, i.e. if $r_i =: r$ for all $i= 1,\ldots ,n$, then the expression in Eq. (3) simplifies to

4 $$\begin{aligned} P_V = \sum _{k= (n+1)/2}^{n} \left( {\begin{array}{c}n\\ k\end{array}}\right) \, r^{k}(1-r)^{n-k} \ . \end{aligned}$$

With the help of Eqs. (3) and (4), we can explore the truth-tracking properties of majority voting. It is well known that $P_V$ in Eq. (4) strictly monotonically increases with *n* and converges to 1 for $r > 0.5$. This is the Condorcet Jury Theorem; $P_V$ in Eq. (3) strictly monotonically increase with *n* and converge to 1 if the average of the $r_i$’s is greater than 0.5.

### The deliberation procedure

Our model is inspired by the movie *Twelve Angry Men*. Here the members of a jury in court meets in a closed room after attending the procedure in the court room. They are allowed to leave the room only after coming up with a consensual verdict. During the procedure, no new evidence comes up (everything was already presented in the court room). However, some people forgot or did not notice certain pieces of evidence. There is also disagreement about the strength of certain pieces of evidence. Initially, the jury members do not know each other at all (they were randomly assembled). They therefore do not know how much weight they can assign to the verdicts of their colleagues. However, during the course of deliberation they get to know each other much better. They see how the others argue, how they criticize the arguments of others, and what they remember of the details of the case. This helps them to better assess how reliable the other group members are and which weight to assign to their verdicts. The deliberation procedure proceeds in several rounds of voting (with discussions in between). It starts with an initial voting in which 11 jurors are for ‘guilty’ and 1 for ‘innocent’. Afterwards a discussion takes place, followed by the next round of voting. The result is now 9:3 and so on until the result of another round of voting is 1:11. The group then convinces the last member in favor of guilty to change his mind, and the movie ends.

The movie inspires our model of deliberation as it presents a clearly structured deliberation process for a situation where all group members have the same knowledge (as they all attended the procedure in court and no new information comes in). The big challenge for modeling this procedure is to specify what happens in between the rounds of voting. Here, people present arguments and criticize each others arguments. This is impossible to do in a model as general and as simple as the one we want to present. To proceed that we are only interested in the result of the deliberation process. We assume that it is the interest of each group member that the probability that the final verdict of the group corresponds to the truth is as large as possible. Each group member wants to to maximize this probability, and to do so it is important that each group member estimates the reliability of the other group members well. Here we assume, as in the previous section, that each group member $a_i$ has a *first order reliability* $r_i$ to make the right judgement. However, the other group members do not know this reliability. They can only estimate it. They will do so on the basis of what the other group members say in the course of deliberation. This suggests that the group members get better and better in assessing the reliability of the other group members. This implies that each group member also becomes better and better to make the right judgement in the various voting rounds provided that they are fairly competent to judge the reliability of the other group members well.

To model this, we assume that each group member has a second order reliability $c_i$ to judge the first order reliability of the other group members. If $c_i =1$, then group member $a_i$ assigns the correct first order reliability $r_j$ to group member $a_j \ne a_i$. (We assume that all group members have perfect access to their own reliability which is an assumption that may be wrong empirically but it can be relaxed easily). If $c_i = 0$, then group member $a_i$ assigns a random reliability from the interval (0, 1) to group member $a_i$. If $c_i \in (0, 1)$, then we follow the procedure specified below which basically assigns a reliability drawn from a more or less broad distribution around $r_i$ (depending on the value of the second order reliability). This reliability weights the verdict of group member $a_j$ in each voting round.

The group members are therefore characterized by two parameters. The first order reliability $r_i$ to make the right decision, and the second order reliability $c_i$ to assess the first order reliability of the other group members. We assume that these two reliabilities are independent. There may be people who have a high first order reliability and a high second order reliability, but there are also people who are good at getting the facts right, but fail to assess the reliability of others. And vice versa. So, without any further knowledge about the particular group we are interested in, we are on the safe side if we assume that the two reliabilities are independent.

We assume that the first order reliability is kept fixed during the course of deliberation. It characterizes, in general terms, how good a certain group member is in making the right judgement. In the course of the deliberation, the quality of the judgement of the group member only goes up because she learns to better weigh the judgements of the other group members. That is, we assume that the second order reliabilities increase in the course of the deliberation because they learn to better judge the reliabilities of the other group members as the deliberation process reveals new information about their reliability (but not about the fact under consideration).

The deliberation procedure we propose then works as follows. The group has to decide on the truth or falsity of a hypothesis H. Each group member assigns a certain probability to H. Then each group member casts a vote on the basis of this probability. Then each group member updates her probability on the basis of the votes of the other group members, weighted according to the estimated reliabilities as explained above. The procedure is iterated, and in each round the second order reliabilities are increased which leads to a more accurate estimation of the reliability of the votes of the other group members. After a number of rounds, this process converges.

One disclaimer before we continue: we call the process we model here a deliberation as it (i) involves the change of belief of the group members in every round and as it (ii) leads to a consensus (as we will see). The model involves several idealizations and black-boxes what happens in between the various rounds of voting. We insist, however, that what happens effectively models a deliberation process.

Let us formalize things now a bit more. First, every group member casts an initial vote, $V^{(0)}_i$ or $\lnot V^{(0)}_i$, for or against the hypothesis in question. We introduce parameters $p^{(k)}_i$ and set $p^{(k)}_i =1$ if $V^{(k)}_i$ and $p^{(k)}_i = -1$ otherwise. These initial votes, for each person, come from an initial probability assignment $P^{(0)}_i(\mathrm{H})$. We assume that group member $a_i$ will initially vote $V_i$ if $P^{(0)}_i(\mathrm{H}) \ge 0.5$ and $\lnot V_i$ otherwise.^3^ This relates to the first order reliabilities in an obvious way, that is, the group member with first order reliability $r_i$ will assign an initial probability greater or equal to 0.5 (and thus vote correctly) with probability $r_i$. Next, every group member $a_i$ estimates the first order reliability $r_j$ of her fellow group members $a_j$, viz.

5 $$\begin{aligned} r^{\left( 0\right) }_{ij} := P^{\left( 0\right) }_i\left( \mathrm{V_j \vert H} \right) = P^{\left( 0\right) }_i\left( \mathrm{\lnot V_j \vert \lnot H} \right) . \end{aligned}$$

**Fig. 1:**
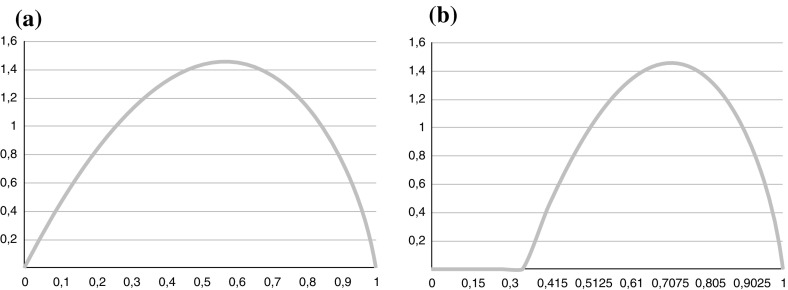
The $\beta$-distribution with parameters $\alpha =2$ and $\beta = 1.625$ corresponding to $r_j=0.75$ and $c_i= 0.6$. **a** $\beta$-distribution on $(0\,\,, \,\,1)$. **b** $\beta$-distribution in (**a**) transferred to the open interval (0.35, 1)

The higher $a_i$’s second order reliability, the better is $a_i$’s assessment of the first order reliability of $a_j$, i.e. the closer is $r^{(0)}_{ij}$ to $r_j$. If the initial second order reliability $c_i^{(0)}=1$, then $r^{(0)}_{ij} = r_j$. If the initial second order reliability $c_i ^{(0)}= 0$, $a_i$ randomly assigns a first order reliability from the interval (0, 1) to $a_j$ (for $j=1,\dots ,n$). If the initial second order reliability is in between the extremes, then agent $a_i$ assigns a reliability $r^{(0)}_{ij}$ to agent $a_j$ by drawing from a $\beta$-distribution whose width is small if $a_i$’s second order reliability is large and whose width is large if $a_i$’s second order reliability is small. To model this, we assume that the estimated first order reliability $r^{(0)}_{ij}$ is calculated from a $\beta$-distribution translated to an interval around $r_j$. The length of this interval is defined by the $c_i^{(0)}$. Higher values of $c_i^{(0)}$ will result in smaller intervals surrounding $r_j$ and thus a more accurate estimation. To do so we consider a $\beta$-distribution with parameters

$$\begin{aligned} \alpha =2 \quad , \quad \beta =\frac{\min \left( 1, r_j - c_i^{\left( 0\right) } +1 \right) -\max \left( 0, r_j + c_i^{\left( 0\right) } -1\right) }{r_{j}-\max \left( 0, r_j + c_i^{\left( 0\right) } -1\right) } \end{aligned}$$

in [0,1] which is then linearly transferred to the interval $\big [ \max (0, r_j + c_i ^{(0)}-1), \min (1, r_j - c_i^{(0)} +1 ) \big ]$. This procedure is illustrated in Fig. 1. The values $\alpha$ and $\beta$ are set such that the $\beta$-distribution has the mode $r_{j}$ after it is transferred to the required interval.

It turns out that our results do not vary much with the value of $\alpha$. What counts is that the $\beta$-distribution has the mode $r_{j}$ after it is transferred to the interval defined by $r_{j}$ and $c_{i}^{(0)}$.

We furthermore assume that the group members become more competent in estimating the first order reliabilities of the other group members. That is, we assume that the second order reliability $c_i^{(k)}$ in round *k* increases linearly as a function of the number of rounds until a maximum value $C_i \le 1$ is reached after *M* rounds. Afterwards, $c_i^{(k)}$ remains constant. Hence,6Note that this is a very simplified (and non-Bayesian) updating process for the second order reliabilities. It effectively models the epistemic effect of the exchange of arguments. We come back to this below.

Using these reliability estimates, each group member $a_i$ calculates the *likelihood ratios* 4

7 $$\begin{aligned} x^{\left( 0\right) }_{ij} := \frac{ P^{\left( 0\right) }_i\left( \mathrm{V_j \vert \lnot H}\right) }{ P^{\left( 0\right) }_i\left( \mathrm{V_j \vert H} \right) } = \frac{1-r^{\left( 0\right) }_{ij}}{r^{\left( 0\right) }_{ij}}, \end{aligned}$$

for $j \ne i = 1,\dots ,n$. The revision process is carried out on the basis of the votes cast by the other group members and their estimated likelihood ratios:

8 $$\begin{aligned} P^{\left( 1\right) }_i \left( \mathrm{H}\right)= & {} P^{\left( 0\right) }_i \left( \mathrm{H \vert Vote^{\left( 0\right) }_1, \dots , Vote^{\left( 0\right) }_{i-1}, Vote^{\left( 0\right) }_{i+1},\dots , Vote^{\left( 0\right) }_n}\right) \nonumber \\= & {} \frac{P^{\left( 0\right) }_i \left( \mathrm{H}\right) }{P^{\left( 0\right) }_i \left( \mathrm{H}\right) + \left( 1- P^{\left( 0\right) }_i \left( \mathrm{H}\right) \right) \, \prod _{k\ne i =1}^n \left( x^{\left( 0\right) }_{ik} \right) ^{p_k} }. \end{aligned}$$

Here $\mathrm{Vote_i^{(0)}}\in \{V_i, \lnot V_i\}$. To derive Eq. (8), we have assumed independence condition9This condition, which is also assumed in the derivation of the Condorcet Jury Theorem (see Eq. (1)) makes sense for a procedure of rational deliberation: The only *cause* for a group members’ verdict is the truth or falsity of the hypothesis in question. The verdicts of the other group members do not have any direct influence on a group members revised verdict. However, the verdicts of the other group members are *evidence* for the truth or falsity of the hypothesis, and a rational group member should take them into account by updating on them (weighted, effectively, with the estimated reliability of the other group members).

Note that the independence condition (9) is assumed to hold in each round of the deliberation process. This requirement is a plausible condition for rational agents, though it may be empirically violated. What our model does, then, is to provide a normative benchmark for the assessment of actual deliberations.

The group members will then vote again and again based on their updated probabilities. As before, a group member votes for the hypothesis if her updated probability is greater than or equal to 0.5, otherwise she votes against it. For example, the second round of deliberation starts with the prior probabilities $P^{(1)}_i (\mathrm{H})$, and everybody repeats updating her probability assignments as before by considering the new votes. As a result, we find that the individual votes converge to a group consensus. Our simulations show that the convergence process is fairly fast. We often need less rounds to arrive at a consensus than the twelve angry men. Therefore the specific value of $C_i$ in Eq. (6) does not matter. We set is to 0.6 in our simulations.

## Truth tracking

In this section, we explore under which conditions the proposed deliberation procedure is truth-tracking. To do so, we distinguish between homogeneous groups (Sect. 3.1) and inhomogeneous groups (Secy. 3.2). In a homogeneous group, all group members have the same first order reliability. In this case, we obtain several analytical results. In an inhomogeneous group, different group members have different reliabilities and results can only be obtained in agent-based simulations. In these simulations we randomly assign reliabilities to the various group members from a uniform distribution and average the results over many runs.

### Homogeneous groups

Let *G* be a homogeneous group of *n* members, i.e. a group whose members have the same first order reliability. This group deliberates on the truth or falsity of the hypothesis *H*. We assume that each group member has access (through some shared history for example) to each others’ first order reliabilities (corresponding to $c_i=1, i=1,\dots ,n$). We furthermore assume that the group members revise their probability assignment for the truth of the hypothesis using the above procedure. Without loss of generality we assume the hypothesis to be true. Then the following theorem holds.

#### Theorem 1

For a homogeneous group G with reliable group members (i.e. for $r > 0.5$), the following three claims hold:

(i) The probability that the group reaches a consensus in finitely many steps increases with the size of the group and approaches 1 as the size of the group increases.
(ii) If the majority of the group members vote correctly in the first round, the subjective beliefs will stabilize on the truth in finitely many steps, i.e. after finitely many steps, each group member assigns subjective probability 1 to the truth of the hypothesis after which the deliberation process will no more change the probability assignments.
(iii) If the majority of the group members vote incorrectly in the first round, the subjective beliefs will stabilize on the wrong belief in finitely many steps, i.e. after finitely many steps, each group member assigns subjective probability 0 to the truth of the hypothesis after which the deliberation process will no more change the probability assignments.

#### Proof

See Appendix 1. $$\square $$

For a homogeneous group *G* with partially reliable members, i.e. a group whose members have a first order reliability $r < 0.5$, the situation is more complicated and the emergence of a consensus depends strongly on the size of the group and the initial probabilities. To see this notice that for $r <0.5$ we will have $x>1$ and thus $x^{\sum _{j=1}^{n}p^{(0)}_j} <1$ if and only if $\sum _{j=1}^{n}p^{(0)}_j < 0$, i.e. if the majority of the group members vote incorrectly in the first round. Using the same argument as in the Condorcet Jury Theorem the chance that the majority of the group members (with first order reliability less than 0.5) will vote incorrectly increases with the size of the group and approaches 1. Thus using the argument in the proof of Theorem 1 if the majority of the group members start with initial subjective probabilities of less than 0.5 for H and hence vote incorrectly in the first round, the probability assignments will increase in the next round and this continues until at some point, say at round *t*, the majority assigns a probability greater than 0.5 for H and thus votes correctly. After this stage the process will reverse and the probabilities will start to decrease since $\sum _{j=1}^{n}p^{(t)}_j > 0$ and thus $x^{\sum _{j=1}^{n}p^{(t)}_j} >1$. If the size of the group, the likelihoods and the initial probabilities are such that at some round *s*-1 the majority assign probabilities less than 0.5 (and thus vote incorrectly) but the probabilities increase in such a way that in round *s* all the probability assignments are above 0.5 then the group reaches a consensus at this round *s*. On the other hand if the probability assignments increase until at some round *s*-1 the majority *but not all group members* assign a probability above 0.5 (so the probabilities decrease in the next round) and in round *s* all probabilities decrease to less than 0.5 then the group will again reach a consensus but this time on the wrong answer. Otherwise the group can oscillate (not necessarily in consecutive rounds) between the case where the majority vote correctly and the case where the majority vote incorrectly. In any case, the subjective beliefs of the group members will not stabilize for partially reliable groups.

#### Theorem 2

For a homogeneous group *G* with partially reliable group members (i.e. for $$ r < 0.5$$), the subjective beliefs of the group members will not stabilize even if the group reaches a consensus.

#### Proof

See Appendix 2. $$\square $$

Notice that in the proof of Theorem 1, the actual value of the likelihood ratio *x* is not relevant. All that matters is whether $x>1$ or $x<1$. This allows for an immediate generalization of these results.

The situation in Theorems 1 and 2 is highly idealized as we assume that the second order reliability is 1, which means that the group members have access to each others’ objective first order reliabilities. In such a context it will be hard to justify the iteration of the deliberation process after the second round. Assuming that group members are able to weight each others’ opinion by the actual objective first order reliabilities there is no room for improvement of such opinions by iteration of the deliberation process more than once. Corollary 1 allows a generalization that makes the iteration of the deliberation process meaningful and extends our results to nontrivial cases where the second order reliabilities are less than 1.

#### Corollary 1

For a homogeneous group G with first order reliability *r*, let the second order reliabilities $c_i$ for $i=1,\ldots ,n$ be less than 1 (so the group members won’t have access to each others’ actual first order reliabilities) but high enough so that the group members can correctly assess whether or not the other group members are reliable, that is let $c_i$ be high enough so that $r_{ij}>0.5$ if and only if $r_j >0.5$ for $j=1,\ldots ,n$. Then the results of Theorems 1 and 2 still hold.

According to Corollary 1, to have the results in Theorem 1 one does not need the agents to know each other’s first order reliability precisely. Rather, one should require the second order reliabilities to be high enough that the agents can correctly distinguish between the reliable and partially reliable members of the group. It is also important to note that this does not need to be the case as the deliberation starts. As the group members become better and better in assessing each other’s first order reliabilities in the course of deliberation (because they listen to each others’ arguments and reasons), the second order reliabilities increase. So even for groups with low second order reliabilities, if the deliberation process continues long enough, the iteration of the deliberation process will improve these second order reliabilities until the assumption of Corollary 1 is satisfied and so the emergence of convergence will be guaranteed.

**Fig. 2:**
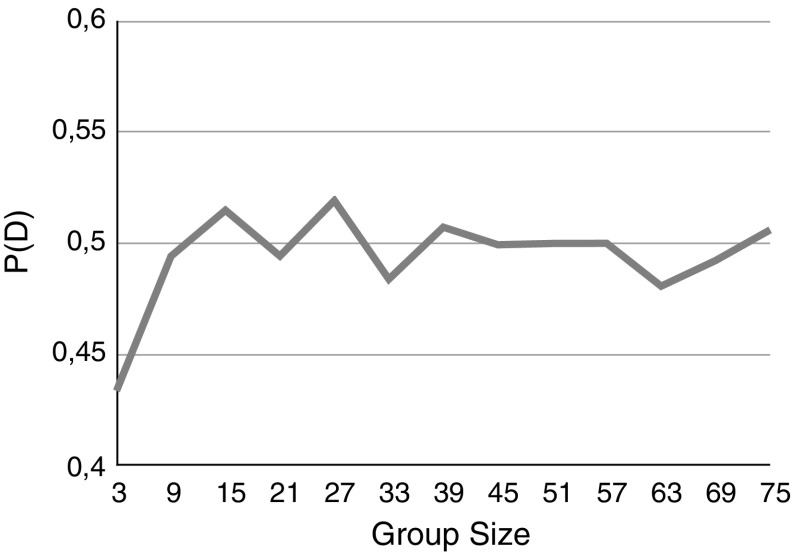
$P_D$ for a homogeneous group as a function of the group size. Each group member has a reliability of 0.4

We conclude this subsection with some simulation results. In Fig. 2 we consider a partially reliable homogeneous group. As we argued above the probability of reaching a consensus on the correct answer can oscillate as the group moves from the case where the majority vote correctly to the case where the majority vote incorrectly.

### Inhomogeneous groups

Let us now consider inhomogeneous groups with second order reliabilities less than 1. Our simulations suggest that the deliberation process also tracks the truth in this case (under plausible conditions). We will also present an illustration of Theorem 2 and the argument preceeding it. To control the noise in the simulation results, we average over $10^{5}$ to $10^{6}$ runs.

**Fig. 3:**
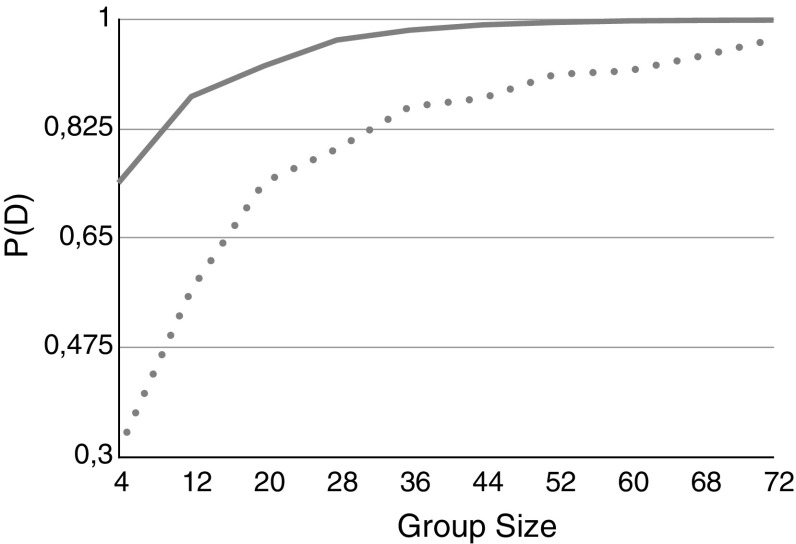
$P_D$ for inhomogeneous groups as a function of the group size. *a* 1/4 of the group members has a reliability of 0.25, the rest has a reliability of 0.7 (*solid line*). *b* 1 / 4 of the group members has a reliability of 0.7, the rest has a reliability of 0.25 (*dotted line*)

Figure 3, shows the probability of tracking the truth in the deliberation as a function of group size. We examine inhomogeneous groups with partially reliable members comprising the minority (Fig. 3a) and the majority (Fig. 3b) of the group members. As the simulation results suggest, in both cases the deliberation tracks the truth for large group sizes. Notice that the group in Fig. 3b has an average first order reliability of less than 0.5, but given the low second order reliabilities the group members do not have access to each others correct likelihood and only estimate these values in a rather large interval.

We conclude that the deliberation procedure (as modeled above) is truth-conducive under similar conditions that hold for the Condorcet Jury Theorem. Note, however, that we do not have an analytical proof for this. The statement is only suggested by the results of the simulations presented here (and many others which we do not show for reasons of space).

## Which procedure is better?

Let us now compare the two procedures. We ask: which procedure is better if our sole goal is to arrive at group decision that is as reliable as possible? We will see that the answer depends on the specific context. There are contexts where the deliberation procedure outperforms majority voting epistemically, and there are contexts where it is the other way around. Which decision procedure is chosen will, of course, also take other considerations into account (as argued in Sect. 1). Section 4.1 focuses on homogeneous groups, and Sect. 4.2 focuses on inhomogeneous groups.

### Homogeneous groups

Let ${\mathcal {X}} =\{ (\pm V_1,\ldots ,\pm V_n) \, \vert \, + V_i =V_i, -V_i =\lnot V_i\}$ be the set of all possible voting profiles for a group of size *n*. A decision rule on ${\mathcal {X}}$ is a function $f: {\mathcal {X}} \rightarrow \{V, \lnot V\}$, that for each voting profile returns a (collective) vote for the hypothesis. As argued in details in Dietrich (2006) the epistemically optimal decision rule is the weighted average where the weights are given by the likelihood ratios. For homogeneous groups this weighted average is reduced to simple majority voting as all group members have the same likelihood ratio and thus the same weight in the averaging process. For groups with very high second order reliabilities the estimated likelihood ratios correspond to the correct values and as one can notice from Theorem 1, for reliable homogeneous groups, the deliberation process will result in a group consensus on the correct (respectively, wrong) answer if and only if the majority of group members vote correct (respectively, wrong) initially. By the same theorem the subjective beliefs will stabilize on the true belief (respectively, wrong belief) if and only if the majority of group members vote correctly (respectively, wrongly) in the beginning. Thus:

#### Proposition 1

For a reliable homogeneous group G with high second order reliabilities, the deliberation process has no epistemic advantage to majority voting and vice versa.

This result is hardly surprising as the weighted average, of which the majority rule for voting is a special case (i.e. all voters get the same weight), has been shown to be epistemically optimal. See Nitzan and Paroush (1982) and Gradstein and Nitzan (1986). That is, if one knows that the group is homogeneous, or if one wants to consider the group to be homogeneous (for political or whatever reasons), then majority voting does best.

The advantage of the deliberation process for these groups, however, is that the group will arrive at a consensus and all group members agree on the collective decision. This is in contrast to majority voting where a minority has to accept the resulting compromise without actually endorsing it. Hence, the advantage of deliberation to majority voting for these groups is merely procedural. For partially reliable homogeneous groups, however, the deliberation process comes with *some* epistemic advantage. For these groups the majority voting is doomed to end with the wrong choice for large groups by the same argument as in the Condorcet Jury Theorem. The deliberation process, however, may converge to the correct answer (depending on the group size and the initial probabilities). One can of course argue that partially reliable homogeneous group are not the right context for comparison of the two procedures since they fall outside the domain in which the majority voting can be considered a justified decision making procedure. Nevertheless we point to how the procedures compare for these groups for the sake of completeness. For reliable homogeneous groups with lower second order reliabilities, however, one would expect the majority voting to preform better than the deliberation procedure. This is so because the deliberation in a reliable homogeneous group *G* with high second order reliabilities is epistemically more efficient than the deliberation in a group $G'$ with the same first order reliabilities as in *G* but with low second order reliabilities. On the other hand, by Proposition 1, voting (in *G* or $G^{\prime }$, notice that it does not matter since group members in *G* and $G^{\prime }$ have the same first order reliabilities) is epistemically as efficient as deliberation in *G* and thus more efficient than deliberation in $G^{\prime }$.

### Inhomogeneous groups

Let us now consider inhomogeneous groups. We have already argued that the deliberation process presents no epistemic advantage over majority voting for homogeneous groups with high second order reliabilities and that for reliable homogeneous groups with low second order reliabilities majority voting does better than our deliberation procedure.^5^ On the other hand for partially reliable homogeneous groups the majority voting is doomed to give the wrong result while the deliberation process *can* end with the consensus on the correct answer as pointed out in the discussion after the Proposition 1. Let us now compare both procedures for various inhomogeneous groups.

In what follows, let $P_D$ and $P_V$ denote the probability of converging to the correct result through deliberation and voting respectively and let

$$\begin{aligned} \Delta = P_D- P_V. \end{aligned}$$

Unless otherwise stated, we plot $\Delta$ as a function of the group size *n*. Unless expressed differently, in all simulations the second order reliability of the group members start from 0.6 and is increased linearly, notice that the second order reliability of 0.6 defines an interval of maximum length 0.8 centered around each $r_j$ (cut at zero or one when necessary) thus allowing for possibly very inaccurate estimations.

**Fig. 4:**
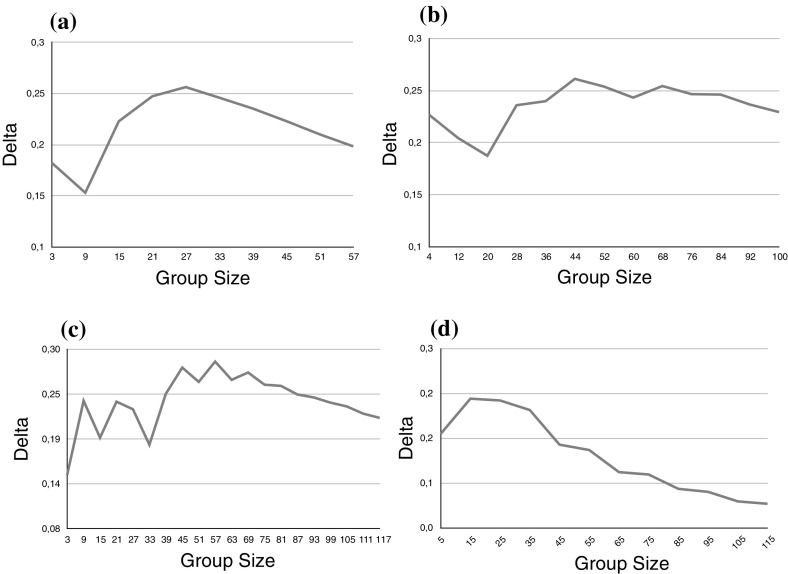
$\Delta$ as a function of the group size. **a** 2/3 of the group has a reliability of 0.7, the rest has a reliability of 0.25. **b** 3/4 of the group has a reliability of 0.6, the rest has a reliability of 0.35. **c** 1/3 of the group has a reliability of 0.8, the rest has a reliability of 0.4. **d** 1/5 of the group has a reliability of 0.95, the rest has a reliability of 0.45

**Fig. 5:**
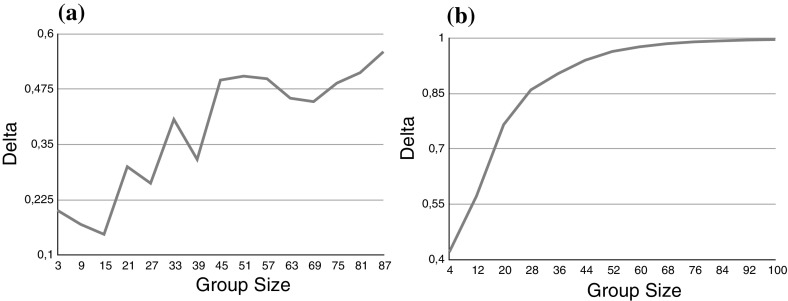
$\Delta$ as a function of the group size. **a** 1/3 of the group has a reliability of 0.75, the rest has a reliability of 0.35. **b** 1/4 of the group has a reliability of 0.8, the rest has a reliability of 0.25.

In Fig. 4a, b, the majority of the group members (2/3 and 4/5, respectively) have a high first order reliability and the rest have a low first order reliability. In Figs. 4c, d the situation is reversed while in all cases the average first order reliability is above 0.5. The simulation results suggest that for inhomogeneous groups the deliberation procedure shows epistemic advantage over majority voting. The difference, however, is more visible for small and medium size groups and becomes smaller as the size of the group increases. This is, of course, not surprising as both $P_V$ (*pace* Condorcet Jury Theorem) and $P_D$ (as suggested by our simulations) coverage to 1. Figure 5 shows the comparison between the deliberation procedure and majority voting for two inhomogeneous groups with average reliabilities of less than 0.5.

The comparison of the deliberation procedure and the voting procedure also depends the second order reliabilities. The probability of the correct choice in deliberation is positively correlated with the second order reliabilities while voting depends only on the first order reliabilities. Thus the difference between deliberation and voting increases for the higher values of second order reliabilities and decreases for lower values.

**Fig. 6:**
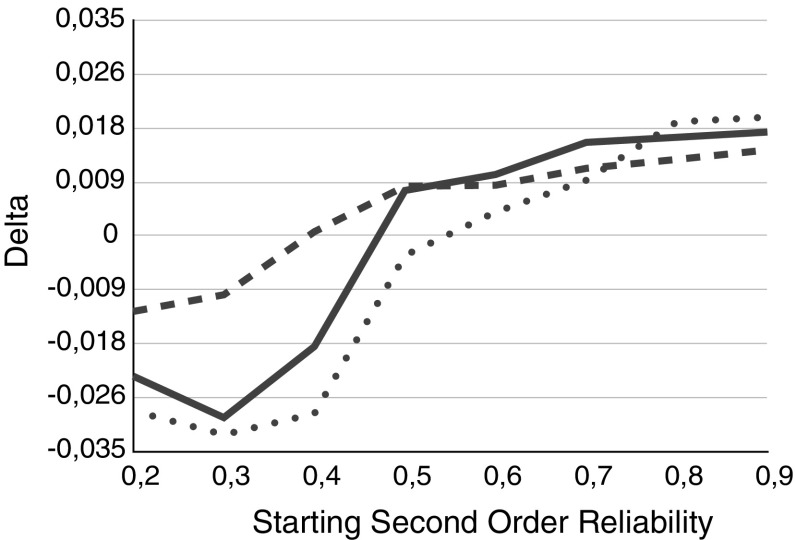
$\Delta$ as a function of the (initial) second order reliability for different group sizes *n*: $n = 15$ (*dotted line*), $n=27$ (*solid line*), and $n=33$ (*dashed line*)

Figure 6 shows the difference between truth tracking in deliberation and voting as a function of the (initial) second order reliability for three different groups sizes ($n= 15, 27$ and 33) with the same distribution of (first order) reliabilities: 2 / 3 of the group has reliabilities of 0.6 and the rest has reliabilities of 0.75. As we can see, the result of the comparison depends highly on the (initial) second order reliabilities. Initial second order reliabilities greater than 0.6, 0.5 and 0.4 make the deliberation procedure epistemically better for groups of size $n =15, 27$ and 33, respectively, while for lower (initial) second order reliabilities the voting procedure performs better.

**Fig. 7:**
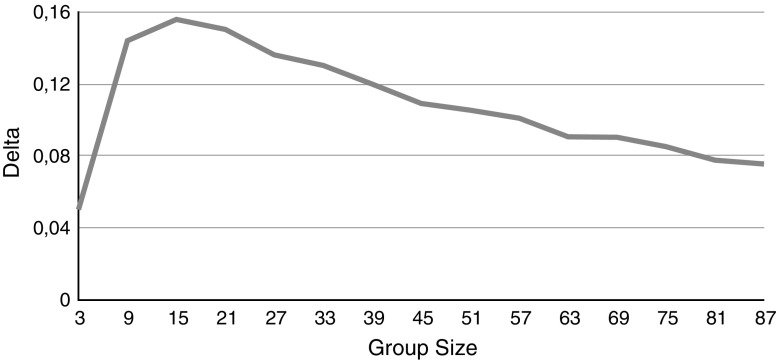
$\Delta$ for a group with only one highly reliable member. One member has a reliability of 0.9, the rest has a reliability of 0.55. The (initial) second order reliability is 0.85

Finally, Fig. 7 shows a group with one highly reliable member where the other group members have near average reliabilities.

Note that the deliberation procedure proposed here does not assume that the first order reliabilities of all group members are known to, say, the chairman of the group. If there were such a person who would have this information, then the weighted average would give the epistemically optimal result, see Nitzan and Paroush (1982) and Gradstein and Nitzan (1986). However, in a real deliberation situation it is often not wanted (for moral or political reasons) that this information is made known, and so a deliberation procedure such as the one proposed here, which relies on the best estimates of the reliabilities of the group members, is the preferred procedure.

## Conclusions

Voting and deliberation are two standard procedures to reach a group decision. The goal of this paper was (i) to present a new Bayesian model for non-strategic rational deliberation, (ii) to study the emergence of consensus and its truth tracking properties, and (iii) to compare this deliberation process with majority voting in terms of their truth-tracking properties. To this end, we proposed a Bayesian model which allows for such a comparison. The model is based on two attributes of the group members: we assumed that each group member has a first order reliability to make the right decision and a second order reliability which specifies how good the group member is in estimating the first order reliability of the other group members. The first order reliability is identical with the reliability used in the modeling of the voting procedure. This identification allows us to compare the two procedures in a meaningful way. Our model focuses on the situation where all group members have the same information about the fact they have to assess (remember the twelve angry men) and where the deliberation process is structures as a sequence of voting procedures. When casting a new vote, each group members takes the verdicts of the other group members in the previous round into account, weighted according to their estimated reliability.

Our model is clearly highly idealized and includes several black boxes. For example, the presentation of arguments and counter-arguments in between the various rounds of voting is modeled effectively by its effect on the assessment of the reliability of the corresponding group member. And so one has to take the results we obtain with a grain of salt. However, the results are plausible and it has to be seen whether more sophisticated models reproduce them.

What are our main results? We have shown that the deliberation process results in a consensus and correctly tracks the truth for groups of large size in the following cases: (i) homogeneous groups with a first order reliability greater than 0.5 and with a high second order reliability. (ii) inhomogeneous groups with average first order reliabilities above 0.5 and with a high (initial) second order reliability. In this sense the deliberation procedure manifests the same epistemic properties as the majority voting while adding the benefit of a group consensus which for groups with average first order reliabilities above 0.5 and high (initial) second order reliabilities will make sure that all group members reach a stable correct belief about the hypothesis in finitely many steps. We furthermore provided simulation results that indicate that the deliberation procedure tracks the truth even in cases that do not fall under the conditions stated in the Condorcet Jury Theorem for majority voting as well as for groups with low second order reliabilities.

Clearly, these results are consequences of our assumptions. But how robust are the results? Do they also hold if we make changes in our deliberation model and relax some of its idealizations? Here are three topics which we would like to address in future work.

First, we want to study the effect of relaxing the independence assumption (9). While it makes sense for voting, the independence assumption is questionable for deliberations from a descriptive, but not from a normative point of view as more and more links between the group members are established in the course of deliberation. This makes the group members (and henceforth also their verdicts) directly dependent on each other. At the end of the deliberation process, when a consensus is reached, it is as if the original assembly of independent individuals has become one homogeneous entity, with all group members endorsing the consensus. The challenge, then, is to model how an increasingly connected social network emerges in the course of the deliberation process and what this entails for the decision-making of the group. We believe that the work presented in this article will be a good starting point for these studies.

Second, we want to study the updating mechanisms for the first order reliabilities. The assumption that the first order reliability remains unchanged during the deliberation makes sense in the context of this article. Note that we are only focusing on contexts where the group members share the same information (they all attended the procedure in court and have no additional knowledge about the case). However, when dealing with situations where different group members have different information at their disposal it is plausible that the first order reliability of the group members changes as a result of the deliberation. It will be interesting to see what taking this into account implies for the main questions we addressed in this article.

Third, it might also be valuable to study more sophisticated updating mechanisms for the second order reliabilities. For example, one can imagine scenarios where the first and second order reliabilities are not independent.

Forth, we have assumed that the agents have no other interests than to track the truth. This is (unfortunately!) an unrealistic assumption in many real deliberations. Are these other interests always negatively interfering with the epistemic goal considered in this article? To address this question, game theoretical models have to be developed.

Addressing these questions requires more detailed models than the one presented here and we hope that our model will be the starting point of many future investigations.

## Appendix 1: Proof of theorem 1

First notice that since all group members have the same reliability $r_i=r$ and the same second order reliability $c_i=1$, the estimated reliabilities in each round will be equal to the actual reliabilities and the likelihood ratio will be the same for all group members in each round, i.e. $x^{(k)}_{ij}=: x = (1-r^{(k)}_{ij})/r^{(k)}_{ij}= (1-r)/r$. So

10 $$\begin{aligned} P^{\left( k+1\right) }_i \left( \mathrm{H}\right)= & {} P^{\left( k\right) }_i \left( \mathrm{H \vert Vote^{\left( k\right) }_1, \dots , Vote^{\left( k\right) }_{i-1}, Vote^{\left( k\right) }_{i+1},\dots , Vote^{\left( k\right) }_n}\right) \nonumber \\= & {} \frac{P^{\left( k\right) }_i \left( \mathrm{H}\right) }{P^{\left( k\right) }_i \left( \mathrm{H}\right) + \left( 1- P^{\left( k\right) }_i \left( \mathrm{H}\right) \right) \, \prod _{j\ne i =1}^n \left( x^{\left( k\right) }_{ij} \right) ^{p^{\left( k\right) }_j} } \nonumber \\= & {} \frac{P^{\left( k\right) }_i \left( \mathrm{H}\right) }{P^{\left( k\right) }_i \left( \mathrm{H}\right) + \left( 1- P^{\left( k\right) }_i \left( \mathrm{H}\right) \right) \, x^{\sum _{j\ne i =1}^{n} p^{\left( k\right) }_j} } \, , \end{aligned}$$

where $p^{(k)}_j \in \{0, 1\}$ is the vote of group member $a_j$ in round *k* and $p^{(k)}_j = 1$ if $Vote^{(k)}_j = V_j$, i.e. if group member $a_j$ has voted (correctly) for the truth of the hypothesis and $p^{(k)}_j = -1$ otherwise. Simplifying this we have $P^{(k+1)}_i (\mathrm{H}) > P^{(k)}_i (\mathrm{H})$ if and only if $x^{\sum _{j \ne i= 1}^{n}p^{(k)}_j} <1$.

The votes in the first round are given by the initial probability assignments that arise from the group members’ reliabilities *r*. This means that group member $a_j$ will start by initially voting correctly, i.e. $p^{(0)}_j =1$ (or equivalently $P^{(0)}_j(\mathrm H) \ge 0.5$) with probability *r* and incorrectly, i.e. $p^{(0)}_j =-1$ (or equivalently $P^{(0)}_j(\mathrm H) < 0.5$) with probability $1-r$. Thus $P^{(1)}_i (\mathrm{H}) > P^{(0)}_i (\mathrm{H})$ if and only if $x^{\sum _{j\ne i=1}^{n}p^{(0)}_j} <1$. Since $r > 0.5$ and $x < 1$, $x^{\sum _{j\ne i=1}^{n}p^{(0)}_j} <1$ if and only if $\sum _{j\ne i=1}^{n}p^{(0)}_j > 0$ that is if the majority of the group members (excluding $a_i$) vote correctly in the first round.

Notice that if the majority of the group members votes correctly in some round, say in round *t*, and if $p^{(t)}_i=-1$ then the majority of the group excluding $a_i$ has voted correctly in round *t* and thus $\sum _{j\ne i=1}^{n}p^{(t)}_j > 0$. If, however, $p^{(t)}_i=1$ it is possible that $\sum _{j\ne i=1}^{n}p^{(t)}_j = 0$ that is when there are exactly the same number of correct and incorrect votes in the rest of the group. In this later case $P^{(t+1)}_i (\mathrm{H}) = P^{(t)}_i (\mathrm{H})$. However, since the probability assignment for any member who has voted incorrectly in round *t* strictly increases, after some finite number of rounds, say *l*, the probability assignment for at least one of these group members, say $a_s$, will increase enough such that $p^{(t+l)}_s=1$ and from then on we have that the number of correct votes in the whole group is at least two more than the number of incorrect ones and thus $\sum _{j\ne i=1}^{n}p^{(t+l)}_j > 0$ for $i=1,\ldots ,n$. Thus for simplicity of notation and without loss of generality we can assume that when the majority of the group votes correctly initially, the number of correct votes is at least two more than the number of incorrect votes. Thus $\sum _{j\ne i=1}^{n}p^{(0)}_j > 0$ for $i=1,\ldots ,n$ and so $P^{(1)}_i (\mathrm{H}) > P^{(0)}_i (\mathrm{H})$ for $i=1,\ldots ,n$. Similarly when we consider the case where the majority of the group members vote incorrectly in the first round we shall assume that the number of incorrect votes is at least two more than the number of correct ones.

In the second round of the deliberation the votes will be casted on the basis of the updated probability assignments. Thus if $P^{(1)}_i (\mathrm{H}) > P^{(0)}_i (\mathrm{H})$ for $i=1,\ldots ,n$ then $\sum _{j \ne i=1}^{n}p^{(1)}_j \ge \sum _{j\ne i=1}^{n}p^{(0)}_j > 0$ since each group member *j* who had voted for the truth of the hypothesis on the basis $P^{(0)}_j (\mathrm{H})$ will still vote the same on the basis of the equal or higher probability $P^{(1)}_j (\mathrm{H})$ while some of the group members who had voted against the hypothesis may change their vote if their subjective probability has been raised to a value above 0.5. Hence from $\sum _{j\ne i=1}^{n}p^{(1)}_j>0$ we have $P^{(2)}_i (\mathrm{H}) > P^{(1)}_i (\mathrm{H})$ for $i=1,\ldots ,n$.

Repeating the same argument the subjective probabilities of the group members (for the truth of the hypothesis) will increases in each round and will be greater or equal to 0.5 in finitely many steps. Thus if the majority of the group members vote correctly in the first round the group will reach a consensus on the correct answer in finitely many steps. If the group members keep repeating the deliberation process (possibly even after the consensus is reached) the probabilities will increase until at some round *t*, we have $P^{(t)}_i(\mathrm H) =1$ for $i=1,\ldots ,n$ after which repeating the deliberation process will no more change the probabilities. This proves part (ii).

By the same argument, if the majority of the group members vote incorrectly in the first round the probability assignments will decreases until after finitely many steps all group members will assign probability zero to $\mathrm H$ and the group will reach a consensus and the subjective beliefs will stabilize (on the wrong belief) and this gives the result for part (iii). Parts (ii) and (iii) will together imply part (i), as it is either the case that the majority have voted correctly in the first round or that the majority have voted incorrectly and in either case the group will reach a consensus in finitely many rounds (on the correct answer and incorrect answer respectively).

If $r \ge 0.5$ then by the Condorcet Jury Theorem the probability that the majority of the group members would vote correctly in the first round (and thus the group reaches a consensus on the correct answer), increases with the size of the group and approaches 1 as the size of the group increases. Similarly if $r <0.5$ by the same argument as in the Condorcet Jury Theorem the probability that the majority of the group members would vote incorrectly in the first round (and thus the group reaches a consensus on the wrong answer), increases with the size of the group and approaches 1 as the size of the group increases. This proves part (i).

## Appendix 2: Proof of theorem 2

Since $r <0.5$ and thus $x>1$, by the argument in the proof of Theorem 1, if the majority of the group members start by voting incorrectly we have that $\sum _{j\ne i=1}^{n}p^{(0)}_j < 0$ and thus $x^{\sum _{j\ne i=1}^{n}p^{(0)}_j} <1$ and the probability assignments increases until the majority will assign a subjective probability above 0.5 to hypothesis at some round *t* (and thus vote correctly) after which $x^{\sum _{j\ne i=1}^{n}p^{(t)}_j} >1$ and the subjective probabilities will decrease and this will repeat. Similarly if the majority start by voting correctly the subjective probabilities will decrease until at some stage the majority will assign a probability less than 0.5 to the hypothesis after which they will vote incorrectly and thus the probability assignments will start to increase, etc.

## Footnotes

1 There are various improvements of the result, such as: (1) The conclusion also holds if one makes the weaker assumption that the average reliability of the voters is greater than 1/2. (2) List and Goodin (2001) generalized the theorem to cases with more than two choice options. (3) Dietrich and Spiekermann (2010) proved a modified version of the Condorcet Jury Theorem where they differentiate between individual dependencies and dependencies on a common cause.

2 See Bohman and Rehg (1997), Cohen (1989), Dryzek (1990), Elster (1998), Fearon (1998), Marti (2006), and Nino (1996).

3 As we disregard strategic considerations in our model, a group member’s vote is only determined by the probability the group member assigns to the corresponding proposition.

4 We follow the convention used in Bovens and Hartmann (2003). Note that $r^{(0)}_{ij} \ge 1/2$ implies that $0 \le x^{(0)}_{ij} \le 1$ and $r^{(0)}_{ij} \le 1/2$ implies that $x^{(0)}_{ij} \ge 1$.

5 Notice that the deliberation procedure gives better results for reliable homogenous groups with high second order reliabilities compared to the same group (same first order reliabilities) but with lower second order reliabilities. This is because the higher second order reliabilities imply more accurate weighting of the votes (with respect to their actual reliabilities). Since for reliable homogenous groups voting does at least as good as deliberation with the assumption of high second order reliabilities, it thus performs better than deliberation with the assumption of low second order reliabilities.
